# Retiring Allowances to Charity Officials

**Published:** 1890-04-26

**Authors:** 


### Passing Topics.

### RETIRING ALLOWANCES TO CHARITY OFFICIALS.

The proceedings publicly reported in reference to the resignation of Mr. Binckes, the secretary of the Royal Masonic School for Boys, have naturally attracted some attention, and if the affairs of an institution of that kind are a little way removed from matter strictly appertaining to the columns of The Hospital, a reference to what has taken place may be not altogether unprofitable, as it serves to illustrate an episode which, sooner or later, enters into the experience of all institutions alike.

It may be remembered a sub-committee recommended some months ago that a retiring allowance of £350 a-year out of the funds of the charity be made to the secretary, who has spent his best years in its service, and whose remuneration presumably has been inadequate, after meeting the ordinary expenditure of life in his station, to allow of making provision for age. The grand committee refused by a narrow majority to accept the recommendation, and it appeared as though a difficult and excessively unpleasant position had been created. Happily this has been disposed of by an immediate payment to the secretary of the sufficient but not extravagant honorarium of £2,500, and, if we understand aright, this sum has been collected especially for the purpose.

The point which appears to claim attention is this: That while the governing body objected to grant their retiring officer an annuity payable out of the funds of the charity they were ready to acknowledge his claim by means of an independent collection. We need not affect ignorance of the fact that some disagreements have occurred among the authorities of the institution, but there is no reason to suppose that these were acute enough to influence this particular decision. Were it otherwise the action of granting a substantial sum by way of testimonial, instead of a pension, would appear more illogical than it even now is.

The present arrangement is not without its recommendations if regarded from the point of view of the institution alone, but we cannot think the precedent a good one. It is not every body of men which can boast the liberality of the Masonic brotherhood, and it might easily come about that the attempt to make a special collection would end in failure. Moreover, a sensitive man might feel a not unnatural indisposition to acquiesce in an arrangement which transformed a pension or grant he could reasonably claim to have earned into a purely compassionate gift.

No doubt the payment of pensions by a society dependent wholly or in great part upon subscriptions, constitutes a burden, but it is one incidental to the carrying out of any permanent undertaking, and after all is more apparent than real, seeing that the incoming official begins with a salary lower than his predecessor obtained. But were the burden more weighty than it is, it could not be evaded without prejudice to the interests of both parties. The chief officer of a charitable institution often possesses a peculiar claim to consideration, inasmuch as the progress and, perhaps, the existence of the charity in question may be due to an expenditure of thought and labour very inadequately represented by the salary paid to him. The acknowledged subscriptions an d donations are by no means the only offerings to charity. Conscientious workers are always devising fresh sacrifices for themselves, and although it is not the fashion to discriminate between payment and remuneration, it is certain that there is no class of work where payment is less commonly remunerative. A man who devotes most if not all of his working years to an object of public utility, ought to be free from anxiety as to what will happen to him when health fails or old age impends; and on grounds of public policy, therefore, we think that the action of the authorities of the Royal Masonic School for Boys is open to mild and friendly protest.

Agricola.

